# Microenvironmental changes induced by placenta-derived mesenchymal stem cells restore ovarian function in ovariectomized rats via activation of the PI3K-FOXO3 pathway

**DOI:** 10.1186/s13287-020-02002-0

**Published:** 2020-11-16

**Authors:** Jong Ho Choi, Jin Seok, Seung Mook Lim, Tae Hee Kim, Gi Jin Kim

**Affiliations:** 1 Department of Oral Pathology, College of Dentistry, Gangneung-Wonju National University, Gangneung-si, 25457 Republic of Korea; 2 Department of Biomedical Science, CHA University, 689, Sampyeong-dong, Bundang-gu, Seongnam-si, 13488 Gyeonggi-do Republic of Korea; 3 Department of Obstetrics and Gynecology, Soonchunhyang University College of Medicine Hospital, Asan, 14584 Gyoenggi-do Republic of Korea

**Keywords:** Placenta-derived stem cells, Ovary function, Oocyte survival, PI3K/Akt, FOXO3a

## Abstract

**Background:**

Translational studies have explored the therapeutic potential and feasibility of mesenchymal stem cells (MSCs) in several degenerative diseases; however, mechanistic studies of the function of these cells have been insufficient. As ovarian failure causes anovulation as well as ovarian steroid hormonal imbalances, the specific aims of this study were to analyze the therapeutic role of placenta-derived MSCs (PD-MSCs) in an ovarian failure ovariectomy (OVX) rat model and evaluate whether PD-MSC transplantation (Tx) improved folliculogenesis and oocyte maturation in the injured ovary through PI3K/Akt and FOXO signaling.

**Methods:**

Blood and ovary tissue were collected and analyzed after various PD-MSC Tx treatments in an ovariectomized rat model. Changes in the expression of folliculogenesis- and ovary regeneration-related genes induced by PD-MSC treatments were analyzed by qRT-PCR, Western blotting, and histological analysis.

**Results:**

The levels of hormones related to ovary function were significantly increased in the PD-MSC Tx groups compared with those in the nontransplantation group (NTx). The follicle numbers in the ovarian tissues were increased along with the increased expression of genes related to folliculogenesis in the PD-MSC Tx groups compared with the NTx groups. Furthermore, Tx PD-MSCs induced follicle maturation by increasing the phosphorylation of GSK3 beta and FOXO3 (*p* < 0.05) and shifting the balance of growth and apoptosis in oocytes.

**Conclusions:**

Taken together, these results show that PD-MSC Tx can restore ovarian function and induce ovarian folliculogenesis via the PI3K/Akt and FOXO signaling pathway.

## Background

Ovarian failure is characterized by the premature loss of ovary follicles and is a common condition in premenopausal women receiving treatment chemotherapy or radiation. Ovarian dysfunction in women can have serious medical consequences, such as blood clots, heart disease, and osteoporosis as well as infertility [1]. For causes other than chemotherapy or radiation, polycystic ovary syndrome (PCOS) in particular is a common female pathology that affects 5~10% of women of reproductive age. These women become infertile because of the aberrant follicle growth of immature follicles [2]. These irregularities in folliculogenesis are further defined by an abnormal relationship between the growth of oocytes and the surrounding granulosa cells [3] in terms of several hormones, including anti-Mullerian hormone (AMH), follicle-stimulating hormone (FSH), and estrogen. The most common treatment for the induction of ovulation is hormone replacement therapy; however, this approach can increase the risk of ovarian and breast cancer [4, 5].

Mesenchymal stem cells (MSCs) have been in the spotlight as a promising cell source to treat a variety of degenerative diseases because of their self-renewal activity, their potential to differentiate into a variety of different cell types, and their immunomodulatory effects. Recently, Johnson et al. showed that BM-MSC transplantation restored oocyte production in mice that lacked ovulation because they had been previously treated with chemotherapy [6]. In addition, BM-MSCs improved ovarian structure and function via the production of VEGF, IGF-1, and HGF in rats with chemotherapy-induced ovarian damage and had the potential to generate immature oocytes and enable long-term fertility in a mouse model treated with chemotherapy [7, 8]. However, there is some controversy regarding the exact role of BM-MSCs in ovarian dysfunction. According to Santiquet et al., transplanted BM-MSCs did not generate new oocytes in a mouse model, although the fertility of female SCID mice post-chemotherapy was improved after transplantation with bone marrow-derived cells [9]. It has been demonstrated that human amniotic fluid stem cells (hAFCs), another cell source, have the potential to differentiate into follicle oocytes and exert a therapeutic effect by restoring ovarian function in mice with chemotherapy-induced sterility [10, 11]. Mesenchymal stem cells derived from the human placenta, namely, human placenta-derived mesenchymal stem cells (PD-MSCs), also have several advantages: (1) there is no donor age dependence, as most cells are at an early stage in development; (2) the cells have high proliferation activity; (3) the cells are easily accessible; (4) the cells are abundant; and (5) the cells are strongly immunosuppressive compared to other mesenchymal stem cells derived from the bone marrow or adipose tissues [12, 13]. Additionally, we reported that PD-MSCs showed high expression of HLA-G compared to that of other MSCs, including BM and adipose MSCs, and that PD-MSCs suppressed the activation of T cells in an in vitro coculture system [14]. Because of these immunosuppressive properties, clinical trials of PD-MSCs are ongoing in several disease types, including certain autoimmune diseases [15–17]. Recently, Yin et al. showed that PD-MSCs have therapeutic benefits in mice with premature ovarian failure because they regulate regulatory T cell activity and cytokines [18, 19].

However, as the mechanisms underlying the therapeutic effect of PD-MSCs have not been fully addressed, we investigated whether PD-MSCs might induce the expression of genes related to ovarian regeneration and whether they retain the ability to restore ovarian function in a rat model of ovarian failure according to various transplantation routes. Furthermore, we scrutinized the signaling mechanisms by which transplanted PD-MSCs are activated and involved in ovarian regeneration in a rat model of ovariectomy.

## Materials and methods

### Animals

All animal experiments were approved by the Institutional Animal Care and Use Committee (IACUC 1500072) of the CHA Laboratory Animal Research Center at Sampyeong-dong in Gyeonggi, Korea. Female 8-week-old Sprague-Dawley rats (Orient Corporation, Seongnam, Gyeonggi, Korea) were used in this study. All rats were housed at two per pathogen-free cage at room temperature (21 °C) with a 12-h light-dark cycle and given ad libitum access to water and standard commercial food.

### Isolation and culture of chorionic plate mesenchymal stem cells

The collection and use of human placentas were approved by the Institutional Review Board (IRB) of CHA General Hospital, Seoul, Korea (IRB07-18). All placental tissues were collected who delivered at term (38 ± 2 gestational weeks) after consent for stem cell research use. Placenta-derived mesenchymal stem cells were isolated from normal chorionic plates of term placentas, as previously described [20]. PD-MSCs were cultured in DMEM/F12 medium supplemented with 10% FBS (Thermo Fisher Scientific, Waltham, MA, USA), penicillin (100 U/ml; Thermo Fisher Scientific), streptomycin (100 μg/ml; Thermo Fisher Scientific), fibroblast growth factor 4 (25 ng/ml; PeproTech, Rocky Hill, NJ), and 1 μg/ml heparin (Sigma-Aldrich, St. Louis, MO, USA) at 37 °C under 5% CO_2_.

### Generation of a rat ovarian failure model by ovariectomy and PD-MSC transplantation

Following acclimatization, the rats were randomly divided into three groups. The NTx group contained ovariectomized (OVX) rats (*n* = 25), the DTx group contained OVX rats directly transplanted with PD-MSCs through the ovary (*n* = 45), and the TTx group contained OVX rats indirectly transplanted with PD-MSCs through tail vein injection (*n* = 45). Ovariectomy was performed in rats of all groups to remove one of the ovaries. All rats were anesthetized by intraperitoneal injection with 250 mg/kg Avertin (Sigma-Aldrich). The surgical site (dorsal area of the pelvis) was disinfected with ethanol, and then, one ovary was accessed and removed by excision through a 1~2-cm skin and muscle incision. After removal of the ovary, the incision was sutured and disinfected with povidone-iodine (Sigma-Aldrich). One week after ovariectomy, PD-MSCs were stained using a PKH67 Fluorescent Cell Linker Kit (Sigma-Aldrich) and injected through the remaining ovary (DTx; 1 × 10^5^ cells) or tail vein (TTx; 5 × 10^5^ cells). Nontransplanted rats were injected with a culture medium. Blood samples were collected weekly and measured for plasma E_2_ using an Estradiol DSL-4400 Radioimmunoassay Kit (Diagnostic System Laboratories, Inc., Webster, TX, USA) before sacrifice. The rats were sacrificed, and ovary tissues were harvested at 1, 2, 3, and 5 weeks.

### Ex vivo culture of ovaries

PD-MSCs were plated in 24-well culture plates at a density of 1 × 10^4^ cells per well and cultured in a modified culture medium [α-MEM (Thermo Fisher Scientific) containing 2% FBS (Thermo Fisher Scientific), 1× penicillin/streptomycin (Thermo Fisher Scientific), 25 ng/ml FGF4 (PeproTech), and 1 μg/ml heparin (Sigma-Aldrich)] at 37 °C under 5% CO_2_. After 24 h, the inserts were precoated with Matrigel (BD Biosciences, San Jose, CA, USA) for 3 h and fitted to each well of a 24-well plate containing PD-MSCs or culture medium alone. The ovary tissues isolated from the normal rats were cut in half using a sterile scalpel blade (JEUNGDO Bio and Plant Co., Ltd., Seoul, Korea) and placed in the upper chamber of the Matrigel-coated insert. The coculture plates were incubated in a 37 °C incubator at 5% CO_2_ for 24, 48, and 72 h.

### Hormone ELISAs

Blood samples were collected from rats in the NTx, DTx, and TTx groups via the retro-orbital technique. The serum was separated from the whole blood by using a serum-separating tube (SST; BD Biosciences). Both the serum samples and the supernatants from the ex vivo cultures were analyzed using ELISA kits for anti-Mullerian hormone (AMH; Elabscience, Beijing, China) and follicle-stimulating hormone (FSH, Abbexa, Cambridge, UK) according to the manufacturer’s instructions. Briefly, an equal volume of a given sample was added to the specific antibody-labeled plates. Then, specific HRP conjugates were added to each well and incubated. Next, the substrates were added to each well and incubated in the dark. After substrate development, the activity of the antibodies was analyzed using a microplate reader (BioTek, Winooski, VT). The concentration of estradiol (E2) was measured by a chemiluminescence immunoassay (CLIA) using a UniCel-DxI800 auto-immunoassay analyzer (Beckman-Coulter, Brea, CA, USA).

### Genomic DNA isolation

Genomic DNA (gDNA) was extracted from the rat ovaries via treatment with proteinase K (QIAGEN, Valencia, CA, USA) and phenol/chloroform (Sigma-Aldrich). The ovary tissues of rats were ground in LN2, and the powdered tissue was then digested overnight at 55 °C in digestion buffer [100 mM Tris pH 8.0 (Abelbio, Jungnang, Seoul, Korea) containing 5 mM EDTA (Bioneer, Daedeok, Daejeon, Korea), 0.2% sodium dodecyl sulfate (SDS; Bioneer), 200 mM sodium chloride (Bioneer), and 0.5 mg/ml proteinase K (Dako, Cambridge, U.K.)]. The supernatants containing gDNA were extracted by using phenol/chloroform (1:1, Sigma-Aldrich) and precipitated with isoamyl alcohol (Sigma-Aldrich) and 0.3 M sodium acetate (Bioneer) at − 20 °C overnight. Next, the gDNA pellet was washed with cold 70% ethanol and eluted using a Tris-EDTA buffer. The gDNA for each sample was analyzed using 1% agarose gel electrophoresis.

### RNA isolation and quantitative real-time polymerase chain reaction analysis

Total RNA was homogenized and extracted from rat ovaries using TRIzol reagent (Invitrogen Thermo Fisher, Camarillo, CA, USA). Total RNA was reverse transcribed into cDNA using Superscript III RNase H reverse transcriptase (Invitrogen) according to the manufacturer’s protocols. Briefly, cDNA transcription of total RNA (500 ng) was first performed with oligo dT (Invitrogen) and dNTP mix (Invitrogen) at 65 °C for 5 min, followed by a second cDNA synthesis performed at 50 °C for an hour and at 72 °C for 15 min with DTT (Invitrogen), RNase out (Invitrogen), Superscript III (Invitrogen), RNase H (Invitrogen), and reverse transcriptase (Invitrogen). cDNA and gDNA were amplified with specifically designed primers (Table S1) and detected using SYBR Green master mix (Roche Diagnostics, Basel, Switzerland) and the Exicycler™ 96 PCR system (Bioneer). The cDNA amplification conditions for qRT-PCR were denaturation at 95 °C for 5 min followed by 40 cycles of 95 °C for 5 s and 59 °C for 30 s. All experiments were performed in triplicate. The expression levels were calculated using the ΔΔCt method after normalization to the mRNA levels of GAPDH as an internal control.

### Western blots

Rat ovary tissues harvested from animal experiments or ex vivo cultures were ground, sonicated, and lysed in protein lysis buffer [RIPA buffer (Sigma-Aldrich) containing a protease inhibitor cocktail (Roche) and phosphatase inhibitors (A.G. Scientific, San Diego, CA, USA)]. Total protein extracts (45 μg) were separated on 8~15% sodium dodecyl sulfate polyacrylamide gels (SDS-PAGE) and transferred to polyvinylidene difluoride membranes (PVDF membranes; Bio-Rad, Hercules, CA, USA). The membranes were blocked with 8% skim milk or 5% BSA (Amresco, Solon, OH, USA) for 1 h at room temperature (RT) and then incubated overnight with primary antibodies (1:1000) at 4 °C, followed by incubation with secondary antibodies (1:20,000) for 1 h at RT using an orbital shaker. Expression was detected with an ECL Advanced Western Blot Detection kit (Amersham, Marlborough, MA, USA) and a ChemiDoc™ XRS+ System (Bio-Rad). The antibodies used in this study included rabbit anti-Nanos3 (Abcam, Cambridge, UK), mouse anti-Nobox (Santa Cruz Biotechnology, Dallas, TX, USA), goat anti-LHX8 (Santa Cruz), rabbit anti-phospho-Akt (Ser473) (Cell Signaling, Danvers, MA, USA), rabbit anti-total-Akt (Cell Signaling), rabbit anti-PI3K (Cell Signaling), rabbit anti-phospho-GSK3beta (Cell Signaling), rabbit anti-p phospho -FOXO3alpha (Cell Signaling), rabbit anti-caspase9 (Abcam), rabbit anti-caspase3 (BD Biosciences), rabbit anti-GAPDH (Invitrogen), HRP-conjugated goat anti-mouse IgG (Bio-Rad), HRP-conjugated donkey anti-goat IgG (Sigma-Aldrich), and HRP-conjugated donkey anti-rabbit IgG (Amersham). All experiments were performed in triplicate. The intensity of each band was quantified with the ImageJ software (NIH, Bethesda, MD, USA).

### Enzyme-linked immunosorbent assay

Both human and rat stem cell factor (SCF) and caspase-3 activities in ovarian tissue lysates and supernatants of ex vivo cultures were measured with SCF ELISA (R&D Systems, Minneapolis, MN, USA) and Caspase-3 ELISA kits (Promega, Gangseo, Seoul, Korea), respectively, according to the manufacturer’s protocols. Additionally, cell necrosis was assessed by the release of lactate dehydrogenase (LDH) into the supernatants of the coculture system. LDH activity was measured using a CytoTox 96 assay system (Promega) following the manufacturer’s protocol. All experiments were performed in triplicate.

### Hematoxylin and eosin staining

To evaluate the number of follicles in rat ovaries after ovariectomy and PD-MSC transplantation, H&E staining was performed. Rat ovary samples were processed in 10% formalin (Millipore, Billerica, MA, USA). Five-micron-thick paraffin sections were stained with H&E and visualized by inverted light microscopy. The total number of follicles less than 100 μm in diameter was counted in at least ten selected nonoverlapping fields.

### Immunostaining

To analyze the expression and localization of LHX8 and Lin-28 in rat ovaries following the transplantation of PD-MSCs, ovary samples were embedded in OCT compound (Fisher Scientific, Pittsburgh, PA, USA). Five-micron-thick cryostat sections were fixed in cold methanol for 10 min and permeabilized with proteinase K (Dako) for 5 min at RT. The sections were blocked with protein block serum-free buffer (Dako) at RT for 30 min and incubated first with goat anti-LHX8 (1:100 dilution, Santa Cruz) at 4 °C overnight and then with rabbit anti-Lin-28 (1:100 dilution, Abcam) in the dark at RT for 2 h. The mixture of secondary antibodies, including Alexa Fluor 488 chicken anti-goat IgG (1:200 dilution, Invitrogen) and Alexa Fluor 594 goat anti-rabbit IgG (1:200 dilution, Invitrogen), was incubated in antibody diluent (Dako) at RT for 1 h, followed by nuclear staining with 4′,6-diamidino-2-phenylindole (DAPI, Vector Laboratories, Burlingame, CA, USA). The stained coverslips were mounted using a mounting solution to avoid light loss. Images were visualized using an Olympus confocal microscope (× 100 magnification) (Olympus, Tokyo, Japan; https://www.olympus-global.com).

### Statistical analysis

Data are represented as the mean and standard error (± SE). Significance was assessed via Student’s *t* test and ANOVA on the SAS software (SAS Institute, Cary, NC, USA), and a *p* value of < 0.05 was used to determine significance (labeled * or #).

## Results

### PD-MSC transplantation increased the serum levels of hormones involved in ovarian function

**Fig. 1:**
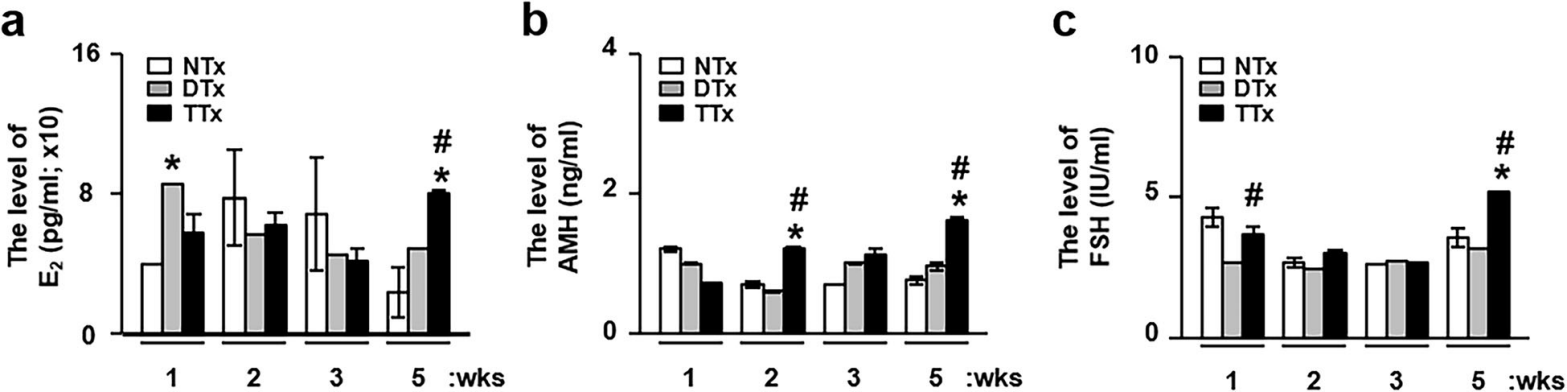
Levels of serum hormones in ovariectomized rats after PD-MSC transplantation. Hormone levels for **a** E2, **b** AMH, and **c** FSH in the serum of ovariectomized rats at 1, 2, 3, and 5 weeks after PD-MSC transplantation were analyzed by chemiluminescence immunoassays and enzyme-linked immunosorbent assays (mean ± SE) (*n* = 5 per group). All experiments were performed in triplicate. Significance at *p* < 0.05 is indicated by an asterisk (*) for NTx vs. others and a number sign (#) for DTx vs. TTx

To investigate whether the effect of PD-MSCs on the levels of hormones related to ovarian function depends on the transplantation route, we measured the levels of estradiol (E2), anti-Mullerian hormone (AMH), and follicle-stimulating hormone (FSH) in the serum of rats in the NTx, DTx, and TTx groups at 1, 2, 3, and 5 weeks after PD-MSC transplantation (Fig. 1). The levels of E2 were increased at 1 and 5 weeks after PD-MSC transplantation in the DTx and TTx groups, respectively, compared to the NTx group (*p* < 0.05); however, these differences were not significant at 2 and 3 weeks. In addition, the levels of E2 in the TTx group at 5 weeks were significantly higher than those in both the NTx and DTx groups (*p* < 0.05, Fig. 1a). Additionally, the production of AMH and FSH in the TTx group at 5 weeks was significantly higher than that in the other groups (*p* < 0.05, Fig. 1b, c). These data suggest that PD-MSC transplantation can increase the levels of hormones related to ovary function in ovariectomized (OVX) rats.

### PD-MSCs engrafted into the ovary increased the number of follicles

**Fig. 2:**
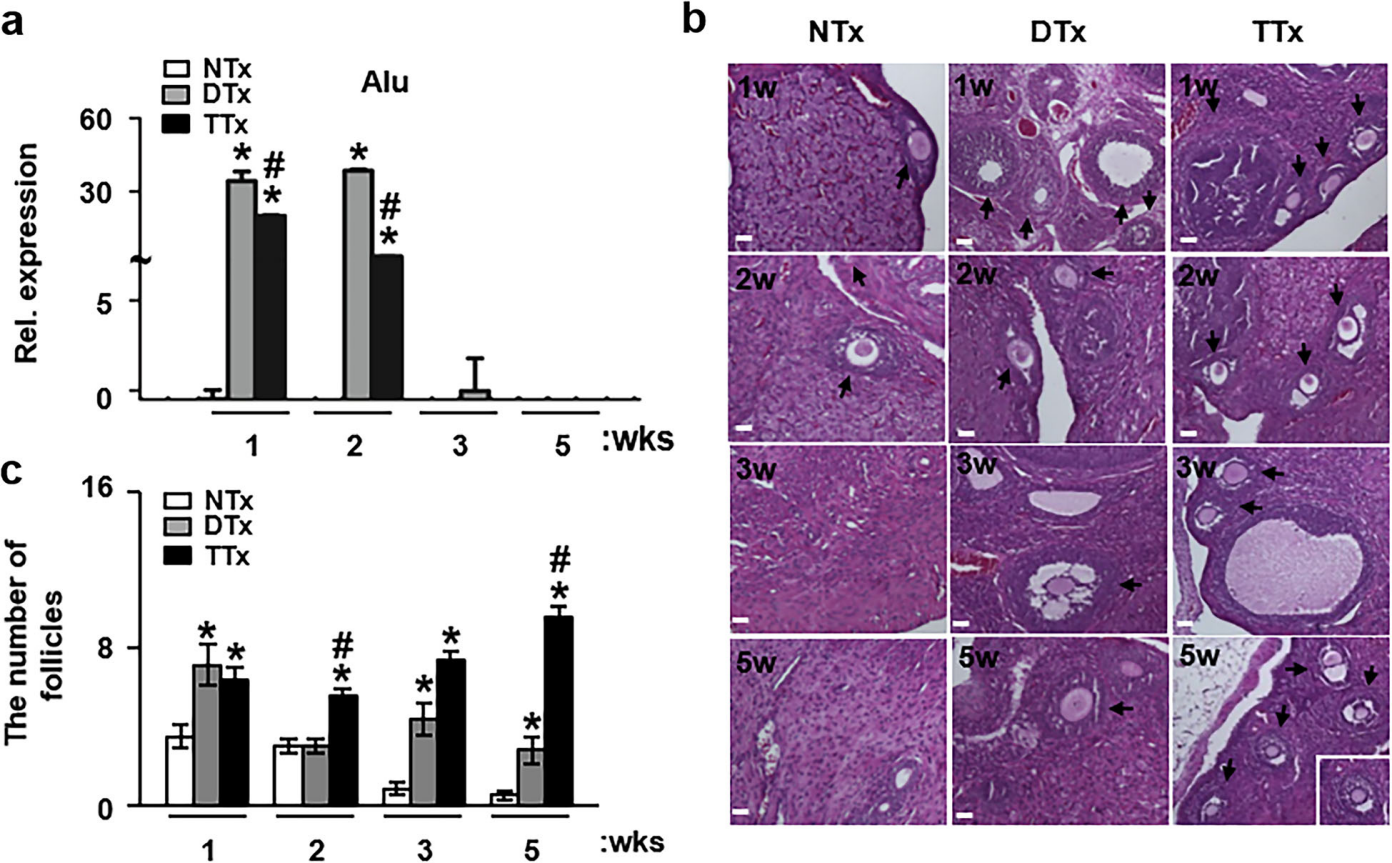
The number of follicles in the ovaries of rats depends on the engraftment of PD-MSCs. **a** The expression of the human Alu gene in ovary tissues of ovariectomized rats at 1, 2, 3, and 5 weeks after PD-MSC transplantation was analyzed by quantitative real-time polymerase chain reaction analysis, normalized to GAPDH as an internal control (mean ± SE) (*n* = 5 per group). **b** Histological staining of the ovaries in ovariectomized rats at 1, 2, 3, and 5 weeks after injection of PD-MSCs was performed with hematoxylin and eosin staining. Scale bars = 50 μm. **c** The number of follicles ≤ 100 μm in the ovary was counted (mean ± SE) (*n* = 3 per group). Significance at *p* < .05 is indicated by an asterisk (*) for NTx vs. others and a number sign (#) for DTx vs. TTx

To confirm that the engraftment of PD-MSCs in a rat model of ovarian failure depends on the transplantation route, we analyzed the expression of the human Alu gene as a human-specific marker in the ovary tissues of rats using qRT-PCR. The expression of human Alu was not detected in the ovary in the NTx group but was strongly expressed in the ovary in the DTx and TTx groups at 1 and 2 weeks post-transplantation of PD-MSCs, although their expression did fade out after 3 weeks post-transplantation of PD-MSCs. Additionally, the expression of the human Alu gene was higher in the DTx group than in the TTx group at 2 weeks post-transplantation (*p* < 0.05, Fig. 2a). These results indicated that PD-MSCs were stably engrafted into the ovary tissues of the rat model by either direct local transplantation or intravenous injection.

We next analyzed whether engrafted PD-MSCs in the ovarian failure model improved the structure or the number of follicles in the ovary tissue of the rats. To quantify the number of follicles, the size of the follicles in an oocyte was defined as below 100 μm and counted following H&E staining. The number of follicles in the ovary tissues in the DTx and TTx groups was significantly higher than that in the NTx group (*p <* 0.05*,* Fig. 2b, c). In addition, the number of follicles in the ovary in the DTx group was highest at 1 week after transplantation, and the number of follicles in the TTx group was highest at 5 weeks post-transplantation (*p* < 0.05, Fig. 2b, c). These results suggest that PD-MSCs can dramatically increase the number of follicles in the OVX rat model in the TTx group; nevertheless, the efficiency of PD-MSC engraftment decreased after 3 weeks post-transplantation with PD-MSCs for both transplantation routes.

### Expression of markers related to folliculogenesis by PD-MSCs was increased in rats with OVX

**Fig. 3:**
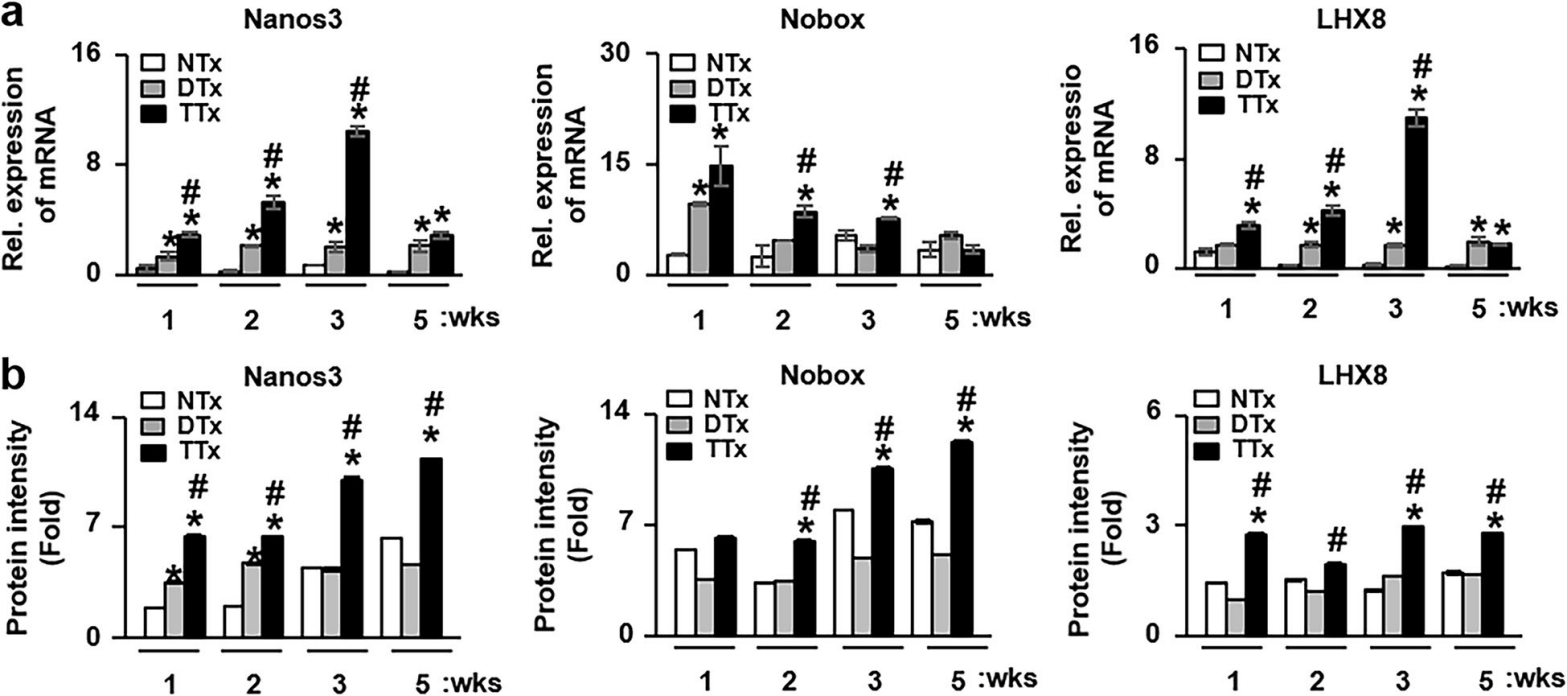
Expression of markers involved in ovarian function after PD-MSC transplantation. **a** The mRNA levels and **b** the protein levels of Nanos3, Nobox, and LHX8 in ovary tissue isolated from ovariectomized rats at 1, 2, 3, and 5 weeks after PD-MSC transplantation were measured by quantitative real-time polymerase chain reaction and Western blot (*n* = 5 per group), respectively. GAPDH was used as a loading control (mean ± SE). Significance at *p* < 0.05 is indicated by an asterisk (*) for NTx vs. others and a number sign (#) for DTx vs. TTx. All experiments were performed in triplicate

It is well known that Nanos3, newborn ovary homeobox (Nobox), and LIM homeobox 8 (LHX8) are essential genes for folliculogenesis in mammalian ovaries [21–23]. To evaluate whether PD-MSCs play a role in folliculogenesis in the ovary via regulation of these genes, we analyzed the expression of the above genes in the ovary tissue from OVX rats using qRT-PCR and Western blotting. The mRNA levels of Nanos3, Nobox, and LHX8 in the ovaries of the rat model were significantly increased by PD-MSC transplantation regardless of the transplantation route (*p* < 0.05, Fig. 3a). The expression levels of these genes were higher in the TTx group than in the DTx group (*p* < 0.05, Fig. 3a). Similarly, the protein expression of Nanos3, Nobox, and LHX8 in the TTx group was dramatically increased compared to that in the NTx and DTx groups; however, there were no differences in the expression of Nobox and LHX8 in the DTx group compared to the NTx group (*p* < 0.05, Fig. 3a and S1). Thus, PD-MSC transplantation can promote the expression of folliculogenesis-related genes, including Nanos, Nobox3, and LHX8, in the ovaries of a rat OVX model.

### Increased LHX8 and Lin28a expression in ovary tissues after PD-MSC transplantation is involved in the balance between oocyte death and survival via activation of the PI3k/Akt and Foxo3 pathways in a rat OVX model

**Fig. 4:**
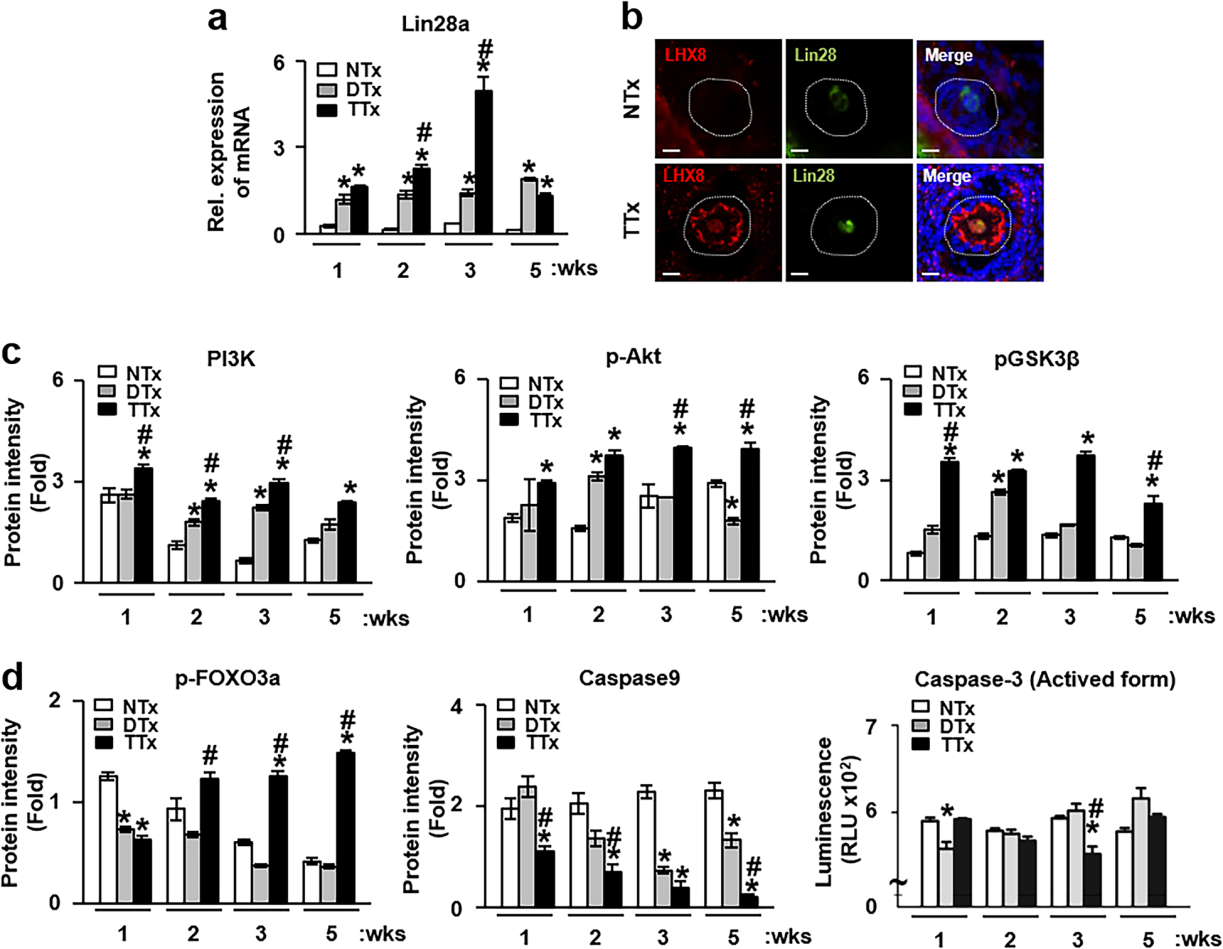
Expression of genes involved in ovarian function in the ovary after PD-MSC transplantation. **a** The expression of Lin28 mRNA in the ovaries of rats at 1, 2, 3, and 5 weeks after PD-MSC transplantation was analyzed by quantitative real-time polymerase chain reaction, with normalization to GAPDH as the internal control (mean ± SE) (*n* = 5 per group). **b** Localization of Lin28 (green) and LHX8 (red) was analyzed by immunofluorescence. The nuclei were stained with DAPI (blue). Scale bar, 50 μm. **c** The intensity of protein expression of PI3K, pAkt, pGSK3β, **d** FOXO3a, and caspase-9 and the activity of caspase-3 were analyzed by Western blotting and enzyme-linked immunosorbent assays and measured with the ImageJ software (mean ± SE) (*n* = 5 per group). GAPDH was used as an internal control. All experiments were performed in triplicate. Significance at *p* < .05 is indicated by an asterisk (*) for NTx vs. others and a number sign (#) for DTx vs. TTx

Recently, Ren and colleagues reported that LHX8 served as a critical oocyte-specific transcriptional factor for primordial follicle activation as well as postnatal folliculogenesis through the LHX8-Lin28a interaction [24]. With this understanding, we checked whether the increased LHX8 expression induced by PD-MSC transplantation affected the expression of Lin28a and whether the PI3K-Akt pathway was activated in the rat OVX model. Our analysis revealed that the mRNA expression of Lin28a was significantly increased in both the DTx and TTx groups compared to the NTx groups for all weeks post-transplantation, and in particular, the expression in the TTx group was higher than that in the DTx group (*p* < 0.05, Fig. 4a). Interestingly, the nuclear levels of Lin28 colocalization with LHX-8 in oocytes were higher after PD-MSC transplantation in the ovarian follicles (Fig. 4b).

We also examined the effect of PD-MSCs on the balance of growth, survival, and death in oocytes in the ovary by investigating the changes in the PI3k/Akt pathway. The expression of PI3K and pAkt in the TTx group was dramatically increased compared to that in both the NTx and DTx groups (*p* < 0.05, Fig. 4c and Fig. S2). In addition, the levels of the well-known PI3K/Akt downstream markers pGSK3β and pFOXO3a were higher in the TTx group than in the NTx and DTx groups (*p* < 0.05, Fig. 4c, d and Fig. S2). On the other hand, representative levels of apoptosis markers, including caspase-3 and caspase-9, were significantly decreased in the DTx and TTx groups compared to the NTx group (*p* < 0.05, Fig. 4d and Fig. S2). These results indicate that transplanted PD-MSCs induce an increase in the expression of Lin28a and LHX8 and influence the balance between the growth and death of oocytes in a rat OVX model by activating the PI3K/Akt and FOXO3 pathways.

### Ex vivo coculture of PD-MSCs with ovarian tissues increases the AMH ovarian reserves, and this is not due to increased maturation of oocytes

**Fig. 5:**
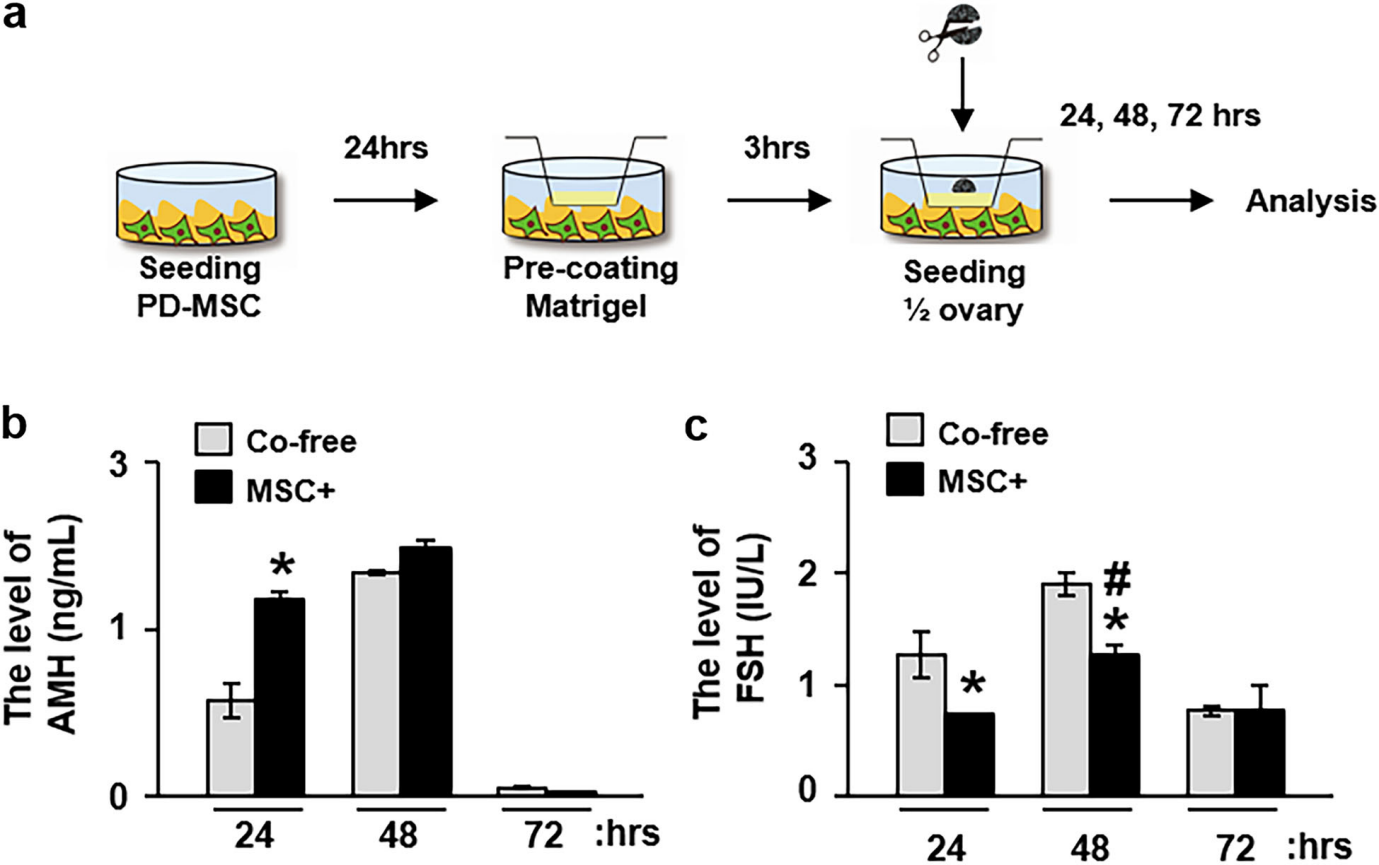
Serum hormone levels in the excised ovaries of rats after coculture with PD-MSCs. **a** Schematic illustration of how the ex vivo coculture system was established. One half of the rat ovary was cultured with or without PD-MSCs for 24, 48, and 72 h. The levels of **b** AMH and **c** FSH in the supernatants at 24, 48, and 72 h after PD-MSC coculture were analyzed by enzyme-linked immunosorbent assay (mean ± SE). All experiments were performed in triplicate. Significance at *p* < 0.05 is indicated by an asterisk (*) for NTx vs. others and a number sign (#) for DTx vs. TTx

To confirm the effect of PD-MSCs on the hormone levels produced by the ovaries, PD-MSCs were cocultured with excised ovarian tissues. The ovarian tissues isolated from rats were cocultured with PD-MSCs on precoated Matrigel plates for up to 72 h (Fig. 5a). The levels of hormones, including AMH and FSH, in supernatants collected at 24, 48, and 72 h after coculture were measured by ELISA. AMH, which is expressed in ovarian granulosa cells and serves as a biomarker for the relative size of the ovarian reserve, was significantly upregulated at 24 h after coculture with the PD-MSCs compared with incubation with medium (*p* < 0.05, Fig. 5b). In contrast, the levels of FSH, which stimulates the growth and recruitment of immature ovarian follicles in the ovary, were decreased (*p* < 0.05, Fig. 5c). There were no differences in the levels of AMH and FSH at 72 h, regardless of whether or not the samples were cocultured with PD-MSCs, and the levels of both of these hormones decreased instead. These findings indicate that cocultivated PD-MSCs induce an ovarian reserve in the excised ovarian tissues through stimulation of AMH produced by ovarian granulosa cells, not recruitment of immature ovarian follicles.

### Coculture of PD-MSCs induces folliculogenesis as well as growth of the ovary via increased stem cell factor ex vivo

**Fig. 6:**
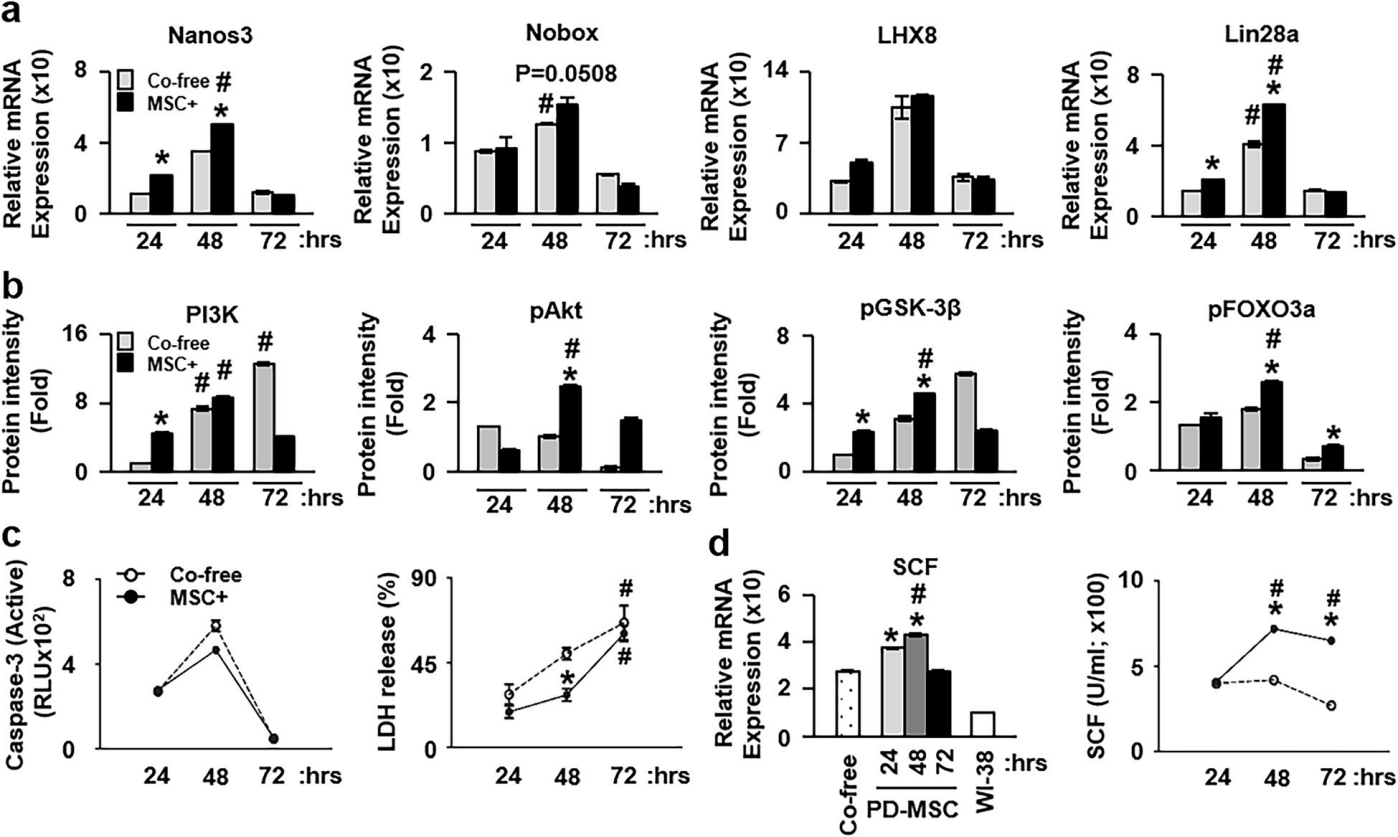
Expression of genes involved in ovarian function in excised rat ovaries after coculture with PD-MSCs. **a** The mRNA expression of Nanos3, Nobox, LHX8, and Lin28a in the ovaries of rats at 24, 48, and 72 h after coculture with PD-MSCs was analyzed by quantitative real-time polymerase chain reaction, with reads normalized to GAPDH as an internal control (mean ± SE). **b** The intensity of PI3K, pAkt, pGSK3β, and pFOXO3a and **c** the activities of caspase-3 in supernatants and in ovary tissues at 24, 48, and 72 h after coculture with PD-MSCs were analyzed by Western blotting and enzyme-linked immunosorbent assays (mean ± SE). The loading control was GAPDH for qRT-PCR and Western blotting. The percentage of LDH release was measured by the LDH assay. **d** mRNA expression (left) and activity (right) of SCF in the ovaries and supernatants at 24, 48, and 72 h after coculture with or without PD-MSCs or WI-38 cells were analyzed by quantitative qRT-PCR and enzyme-linked immunosorbent assay, normalized to GAPDH as an internal control (mean ± SE) All experiments were performed in triplicate. Significance at *p* < 0.05 is indicated by an asterisk (*) for NTx vs. others and a number sign (#) for DTx vs. TTx (± SE)

Here, we demonstrated the increased mRNA expression of Nanos3, Nobox, LHX8, and Lin28a in ovarian tissues cocultured with PD-MSCs ex vivo; this is in line with the ability of transplanted PD-MSCs to induce the expression of several factors related to folliculogenesis in vivo in the rat OVX model. Generally, the mRNA expression levels of Nanos3, Nobox, LHX8, and Lin28a were more significantly increased at 48 h, and the mRNA expression levels of Nanos3 and Lin 28a were higher at 24 and 48 h after coculture with PD-MSCs than in the coculture-free groups (*p* < 0.05, Fig. 6a). These data further support the previous results that the transplantation of PD-MSCs increases folliculogenesis by enhancing the expression of Nanos3, Nobox, and LHX8. Similar to the positive effect on folliculogenesis, the levels of PI3K and pAkt at 24 and 48 h, respectively, were significantly increased in the excised ovarian tissues after coculture with PD-MSCs compared to the coculture-free group (*p* < 0.05, Fig. 6a).

Furthermore, the levels of the PI3K/Akt downstream markers pFOXO3a and pGSK3β were significantly higher in the ovarian tissues at 48 h after coculture with PD-MSCs than in the coculture-free groups (*p* < 0.05, Fig. 6b and Fig. S3). Coculture with PD-MSCs reduced cell death from apoptosis (from active caspase-3 levels) and necrosis (LDH levels) in ovarian tissues compared to those of the coculture-free group, although the frequency of cell death caused by necrosis was increased for all ovarian tissues at 72 h (*p* < 0.05, Fig. 6c). These findings indicate that PD-MSCs trigger the activation of FOXO signaling in ovarian tissues, resulting in the induction of folliculogenesis as well as ovary growth. It is known that the interaction between Kit and stem cell factor (SCF, also known as Kit ligand) on granulosa cells triggers the activation of the PI3K/Akt signaling pathway in ovarian tissues [25]. As such, we checked the expression of SCF in ovarian tissues cocultured with PD-MSCs. The mRNA levels of SCF were significantly increased in ovarian tissues cocultured with PD-MSCs compared to those of the coculture-free group and the group cocultured with WI-38 human fibroblasts at 24 and 48 h (*p* < 0.05, Fig. 6d, left panel). Additionally, the protein levels of SCF in the culture supernatants from cocultures with PD-MSCs were significantly increased at 48 and 72 h compared to those in the coculture-free group (*p* < 0.05). Otherwise, their concentrations were decreased in the supernatant of cocultures with WI-38 cells as a negative control (Fig. 6d, right panel). Moreover, we analyzed the PD-MSC-derived human SCF concentration in the supernatant of the ex vivo experiment. Interestingly, the human SCF concentration in the supernatant with PD-MSCs part in co-cultivation with the ovary tissues was increased at 24, 48, and 72 h (*p* < 0.05, Fig. S4a). Also, the human SCF concentration in the supernatant with ovary tissues part after the PD-MSC cocultivation was increased at 24 and 72 h than compared to those of coculture free groups (*p* < 0.05, Fig. S4b). Therefore, this data showed that PD-MSCs secreted human SCF cytokine during ex vivo for 72 h. These findings indicate that coculture PD-MSCs induced SCF expression in the excised ovarian tissue, leading to the activation of PI3K/Akt and FOXO3 signaling and a reduction in apoptosis levels.

## Discussion

Ovarian failure can result from hereditary conditions or from exposure to various agents, such as those used for cancer therapy. The factors that result in infertility are abnormal ovulation, anovulation (failure to ovulate), hyperandrogenism, and metabolic abnormalities in women of childbearing age, which manifest during menopause and cause various medical problems in the middle years of a woman’s life [26]. Hormone replacement therapy (HRT) with recombinant hormones such as gonadotrophins to address ovarian failure, however, is often associated with side effects that include overproduction of follicles, with manifestations including ovarian hyperstimulation syndrome and multiple pregnancies [27, 28]. To overcome these medical hurdles, various approaches are being developed, including cell-based therapy with stem cells for functional regeneration or enhancement of the ovaries in ovarian failure patients. Recently, Takehara et al. showed that adipose-derived MSCs promoted the restoration of ovary function in an ovary failure model by decreasing the levels of inflammatory cytokines [29]. Wang et al. demonstrated that granulosa cells were affected by transplanted MSCs, as these cells helped restore folliculogenesis in the chemotherapy-induced ovarian failure mouse model [30]. Nevertheless, there are still ambiguities concerning the therapeutic mechanisms of MSC transplantation in ovarian failure models.

MSCs are attractive therapeutic agents, not only because of their potential for self-renewal and differentiation activities but also because MSCs provide the proper microenvironment “niche” to enhance the regeneration of cells or tissues damaged by cancer therapy through various signaling pathways and cellular events [31–33]. Recently, placenta-derived MSCs, which are a representative source of fetal stem cells, have become regarded as an alternative to bone marrow-derived MSCs in potential medical applications. In preclinical studies with various animal models, PD-MSCs have already demonstrated therapeutic potential for degenerative diseases and are currently being tested for such applications in clinical trials worldwide. Further studies, however, on the fundamental mechanisms underlying their therapeutic efficacy are needed.

Previously, we demonstrated that PD-MSCs rapidly induced an increase in the levels of E2 hormone and led to changes in the expression of genes related to folliculogenesis in a rat OVX model when 3D spheroids of PD-MSCs were directly transplanted into the rats, although the precise mechanism of these transplanted cells was not known [34]. We next focused on identifying the most effective transplantation routes to maximize the therapeutic effect of PD-MSCs and investigated their effect on functional improvements, including folliculogenesis and oocyte growth, through PI3K/Akt and pFOXO signaling in a rat model of ovariectomy. Interestingly, PD-MSC engraftment via tail vein transplantation (TTx) showed a dramatic increase in the number of follicles compared to those of the other groups until 5 weeks post-transplantation, although their engraftment was not detected after 2 weeks (*p* < 0.05, Fig. 2). These findings suggest that TTx is a safe and optimal transplantation route for PD-MSCs and that engrafted PD-MSCs disappear in a short period after triggering folliculogenesis and changing the microenvironment in the ovarian tissues in the rat model.

Alteration of the microenvironment niche by stem cells is an important factor required to understand the therapeutic mechanism of transplanted stem cells as well as to enhance their therapeutic effects [35]. In particular, in stem cell therapy, the cell-to-cell interactions and the cross-talk between stem cells and endogenous cells in the target tissues are critical for restoring and regenerating tissues in regenerative medicine [36]. In the present study, we confirmed that PD-MSC transplantation induces an increase in the expression of folliculogenesis-related genes (e.g., Nanos3, Nobox, and LHX8), including an RNA-binding protein, Lin28a, which is involved in the regulation of many microRNAs [37, 38]. Lin28a blocks the production of the mature let-7 microRNA in mouse embryonic stem cells by binding to the let-7 pre-microRNA and thus regulating the self-renewal activity of stem cells [39]. In addition, Lin28a is a well-known regulator of primordial germ cell development in human ovary development through the Akt/mTOR pathway [40]. Ren et al. also showed that LHX8 directly binds to Lin28a, resulting in increased Lin28a expression, as it regulates primordial oocyte activation [24]. Our data indicate that PD-MSC transplantation induces an increase in the expression of LHX8 as well as Lin28a in a rat model. These findings point to the increased LHX8/Lin28a signaling induced by PD-MSCs stimulating folliculogenesis through activation of PI3K/Akt signaling in the rat OVX model.

During mammalian oogenesis, the phosphatidylinositol-3-kinase (PI3K) signaling pathway is a critical regulator of survival, as the loss or activation of follicle development is regulated by several components of the pathway, including Akt, GSK3α, and GSK3β [25], and in particular, the PI3K-PTEN circuit governs follicle activation by contributing to the initiation of oocyte growth [41]. Recently, the oocyte-specific deletion of PTEN led to the stimulation of PI3K activation of Akt phosphorylation and resulted in FOXO3 hyperphosphorylation and nuclear translocation in oocytes, leading to follicle activation [42]. Thus, the PI3K pathway and FOXO3a may be important for oocyte growth as well as follicle activation. FOXO3, which is a FOXO family member, is related to female fertility by regulating folliculogenesis both in oocytes and in somatic granulosa cells and determines oocyte apoptosis or growth and can lead to follicle activation [43].

The FOXO proteins have a “Janus face” for folliculogenesis according to their phosphorylation status and localization in the oocyte. For example, overexpression of FOXO3a in the oocyte nucleus can lead to apoptosis of oocytes by upregulating caspase-3 and caspase-8, proapoptotic proteins, and Bim, FasL, and p27KIP1, which are their key downstream mediators [44]. On the other hand, when FOXO3 is phosphorylated at an Akt site, it is exported from the nucleus to the cytoplasm, resulting in follicle activation [42]. Despite the importance of FOXO3a in ovarian function, the link between therapeutic MSCs and modulation of FOXO3a localization and levels has not been reported. Based on this knowledge, we investigated the effect of PD-MSCs on growth and folliculogenesis in ovarian tissues in a rat model and confirmed that PD-MSCs could restore ovarian function by promoting oocyte growth and inhibiting apoptosis in vivo and ex vivo in the coculture system via the PI3K/Akt/FOXO signaling pathway (Figs. 4 and 6).

In addition, the SCF-PI3K-Akt-FOXO3a-mediated cascade induced by PD-MSCs may play an important role in oocyte growth and follicular activation. In particular, SCF (Kit ligand (KL)) produced by granulosa cells activates the PI3K pathway and serves as an intraoocyte signal via the oocyte-surface receptor Kit [45]. In the absence of SCF, interestingly, FOXO3a induces oocyte apoptosis rather than oocyte growth via the PI3K/Akt pathway, and thus, the SCF from granulosa cells regulates oocyte growth and follicular development by activating the PI3K-Akt-GSKβ pathway in oocytes [25, 46]. In a previous report, increased SCF induced by PD-MSCs activated the PI3K expression, resulting in the upregulation of PI3K-SCF-c-Kit signaling and promoting the self-renewal ability of PD-MSCs through mTOR phosphorylation under hypoxic conditions [47]. This implies that increased SCF induced by PD-MSCs can act as a signal for oocyte growth rather than cell death through PI3K/Akt/FOXO signaling and that PD-MSCs modulate this pathway to restore folliculogenesis and address ovarian dysfunction in a rat OVX model. This is the first report of the molecular mechanism underlying the therapeutic effect of PD-MSCs in restoring ovarian function in a rat OVX model.

## Conclusion

**Fig. 7:**
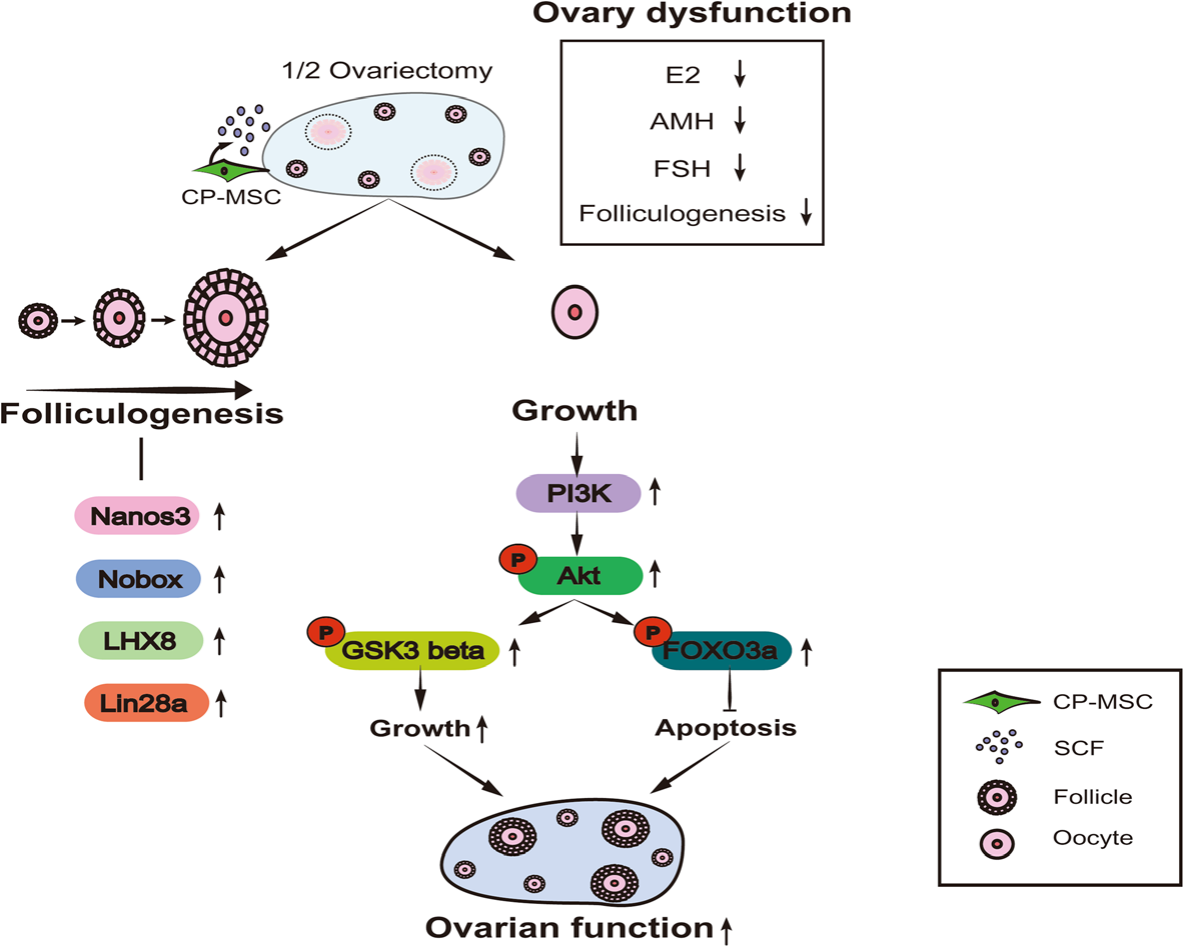
Summary of the ability of PD-MSCs to restore ovary function in the ovariectomized model. PD-MSCs promote folliculogenesis in the ovary, as indicated by the upregulation of pathway markers, including Nanos3, Nobox, LHX8, and Lin28a. Additionally, SCF expressed in PD-MSCs dramatically increased pGSK3β, which is involved in oocyte growth, and decreased oocyte apoptosis by inducing pFOXO3a expression via the PI3K-pAkt pathway

In summary, our results indicate that PD-MSC transplantation can restore ovarian function by promoting folliculogenesis through the upregulation of Nanos3, Nobox, LHX8, and Lin28a. The therapeutic effect could be accelerated by the SCF secreted from PD-MSCs capable of promoting oocyte survival via the PI3K/Akt/FOXO pathway. These results suggest that PD-MSCs can restore ovarian function by regulating folliculogenesis and oocyte growth through the PI3K-FOXO3a pathway (Fig. 7). Our findings suggest that MSC therapy using PD-MSCs can restore ovarian function and be used as an alternative therapy for patients with ovarian dysfunction, including PCOS and infertility. In addition, this work offers new insights into MSC-based therapeutic mechanisms in reproductive diseases.

## Supplementary Information

**Additional file 1 **: **Supplementary Table 1.** Primers used in the present study for qRT-PCR analysis.

**Additional file 2 **: **Figure S1.** Expression of genes involved in folliculogenesis in ovary after PD-MSC transplantation. The expression of protein Nanos3, Nobox and LHC8 in ovary tissue isolated from OVX rats at 1, 2, 3 and 5 weeks after PD-MSCs transplantation were analyzed with Western blot (*n*=5 per group). GAPDH was used as a loading control. All experiments were performed in triplicate.

**Additional file 3 **: **Figure S2.** Expression of gene involved in proliferation in ovary after PD-MSC transplantation. The expression of protein of pAkt, PI3K, pGSK3β, pFOXO3a and capspase-9 in ovary tissue isolated from OVX rats at 1, 2, 3 and 5 weeks after PD-MSCs transplantation were analyzed by Western blot (*n*=5 per group). GAPDH was used as an internal control. All experiments were performed in triplicate.

**Additional file 4 **: **Figure S3.**

**Additional file 5 **: **Figure S4.**

## Data Availability

The data that support the findings of this study are available from the corresponding author upon reasonable request.

## Abbreviations

MSCs: Mesenchymal stem cells
PD-MSCs: Human placenta-derived mesenchymal stem cells
OVX: Ovariectomy
GAPDH: Glyceraldehyde-3-phosphate dehydrogenase
Nobox: Newborn ovary homeobox
LHX8: LIM homeobox 8
PI3K: Phosphoinositide 3 kinase
FOXO3: Forkhead box O3
DAPI: 4′,6-Diamidio-2-phenylindole
AMH: Anti-Mullerian hormone
FSH: Follicle-stimulating hormone
LDH: Lactate dehydrogenase
SCF: Stem cell factor
