# Supplementary material for: Microenvironmental changes induced by placenta-derived mesenchymal stem cells restore ovarian function in ovariectomized rats via activation of the PI3K-FOXO3 pathway
Source: Stem Cell Res Ther. 2020 Nov 16;11:486. doi: 10.1186/s13287-020-02002-0 (PMC7667861; doi:10.1186/s13287-020-02002-0)
Supplement: Supplementary file 1 — Additional file 1 : Supplementary Table 1. Primers used in the present study for qRT-PCR analysis. [file 13287_2020_2002_MOESM1_ESM.docx]

**Supplementary Information**

**Supplementary Table 1**

Primers used in the present study for qRT-PCR analysis

| **Genes** | **Primer sequences** | **Tm (℃)** |
| --- | --- | --- |
| Nanos3 | F: CTC TGC ATG AGG AAG AGG AGC C  R: GGA CTG ATA GAT CGC ACG AGA | 60 |
| Nobox | F: AGC CAG TGC AGA TCT GCA CC  R: TGT CAC TGC CAG GAA CAT CCC TC | 60 |
| Lhx8 | F: GTA TCA CTT GGC TTG CTT  R: ATT ACC GTT CTC CAC TTC | 60 |
| Alu | F: CTG GGC GAC AGA ACG AGA TTC TAT  R: CTC ACT ACT TGG TGA CAG GTT CA | 60 |
| Lin28a | F: CCC GGT GGA CGT CTT TGT G R: CAT TGC CTC ACC CTC CTT GA | 60 |
| GAPDH | F: GGA AAG CTG TGG CGT GAT  R: AAG GTG GAA GAA TGG GAG TT | 60 |

**Supplementary figure legends**

**Fig. S1** Expression of genes involved in folliculogenesis in ovary after PD-MSC transplantation. The expression of protein Nanos3, Nobox and LHC8 in ovary tissue isolated from OVX rats at 1, 2, 3 and 5 weeks after PD-MSCs transplantation were analyzed with Western blot (n=5 per group). GAPDH was used as a loading control. All experiments were performed in triplicate.

**Fig. S2** Expression of gene involved in proliferation in ovary after PD-MSC transplantation. The expression of protein of pAkt, PI3K, pGSK3β, pFOXO3a and capspase-9 in ovary tissue isolated from OVX rats at 1, 2, 3 and 5 weeks after PD-MSCs transplantation were analyzed by Western blot (*n*=5 per group). GAPDH was used as an internal control. All experiments were performed in triplicate.
