# Supplementary figures and images for: Microenvironmental changes induced by placenta-derived mesenchymal stem cells restore ovarian function in ovariectomized rats via activation of the PI3K-FOXO3 pathway
Source: Stem Cell Res Ther. 2020 Nov 16;11:486. doi: 10.1186/s13287-020-02002-0 (PMC7667861; doi:10.1186/s13287-020-02002-0)

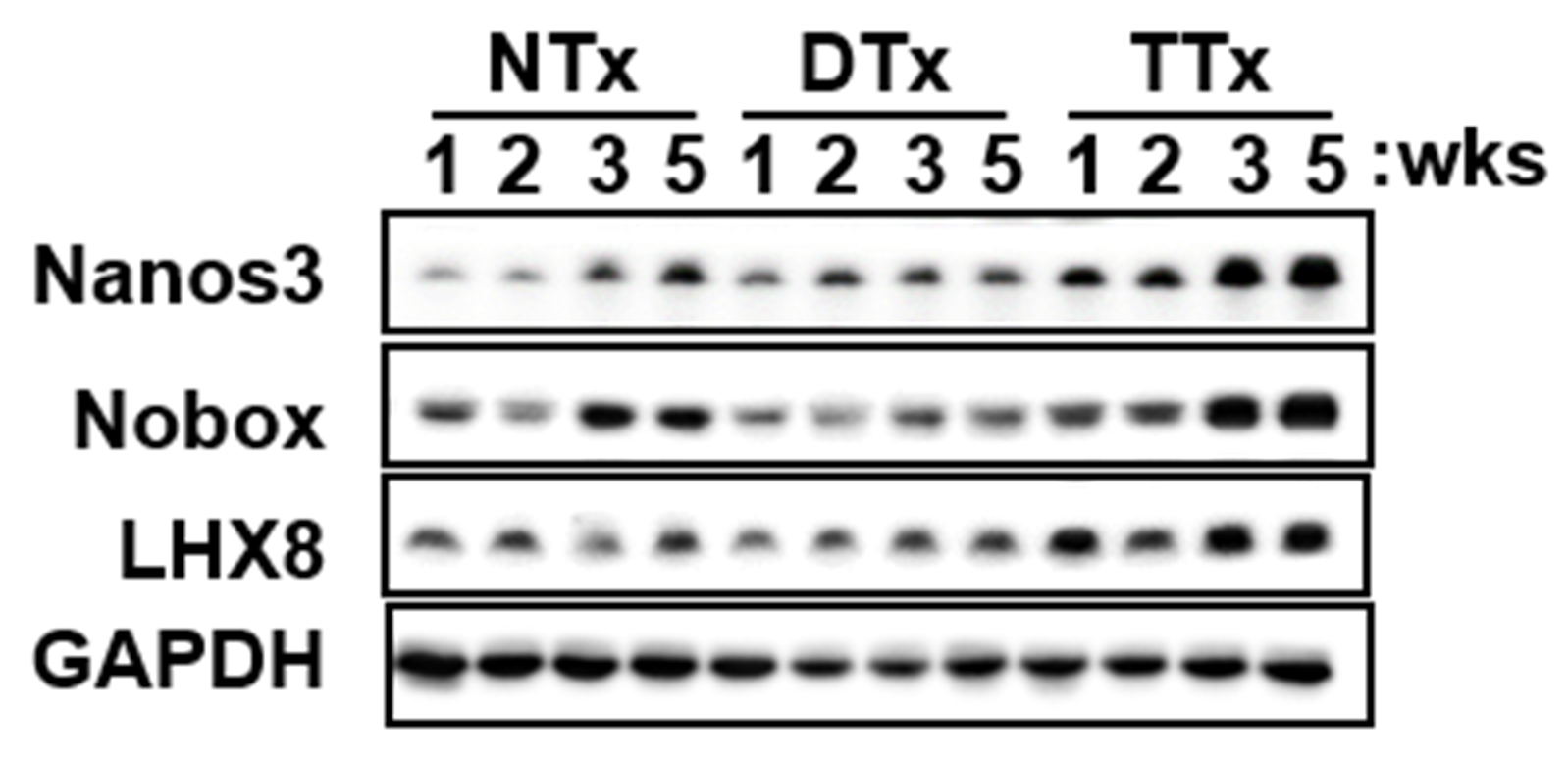

Supplement: Supplementary file 2 — Additional file 2 : Figure S1. Expression of genes involved in folliculogenesis in ovary after PD-MSC transplantation. The expression of protein Nanos3, Nobox and LHC8 in ovary tissue isolated from OVX rats at 1, 2, 3 and 5 weeks after PD-MSCs transplantation were analyzed with Western blot (n=5 per group). GAPDH was used as a loading control. All experiments were performed in triplicate. [file 13287_2020_2002_MOESM2_ESM.tif]

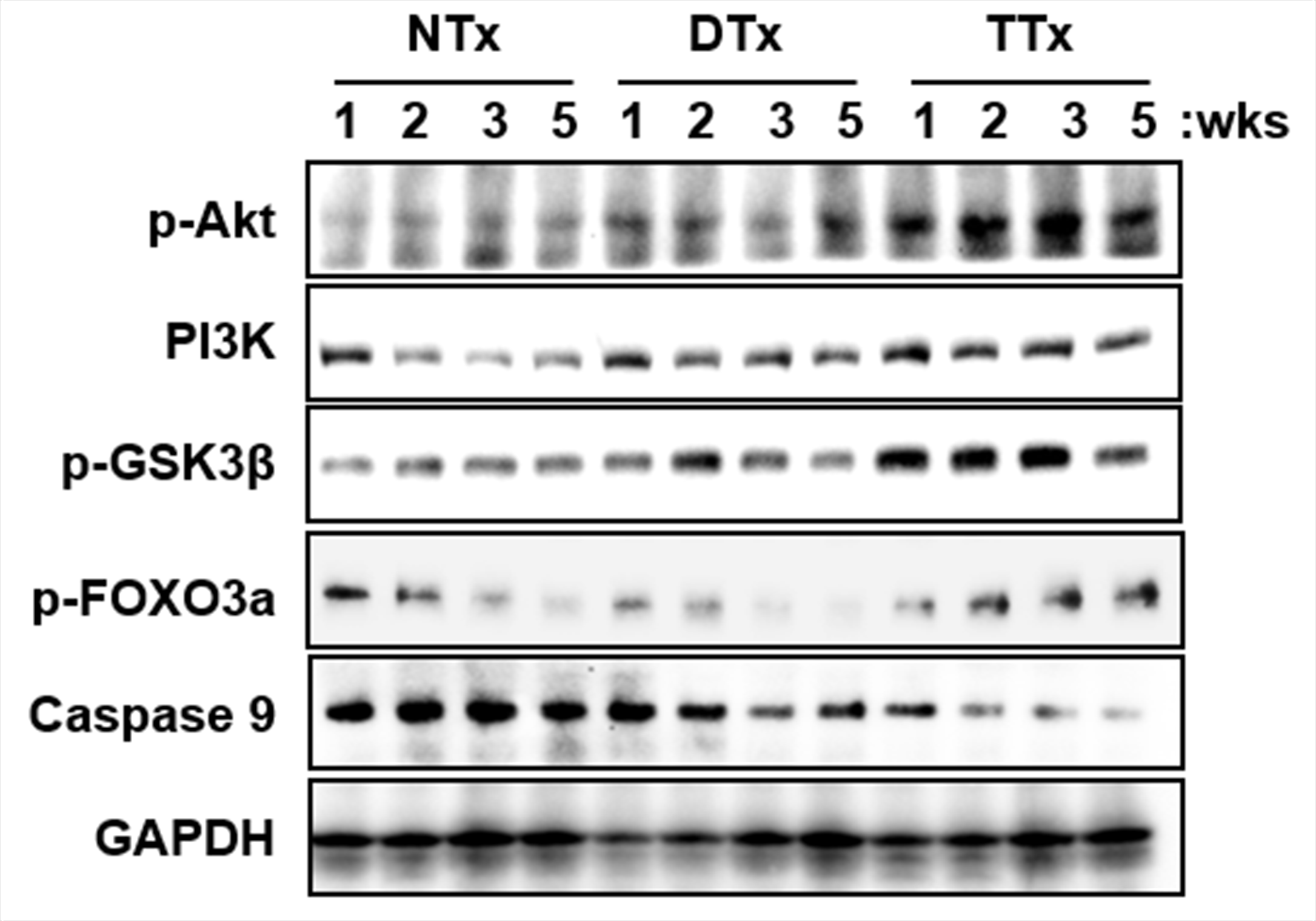

Supplement: Supplementary file 3 — Additional file 3 : Figure S2. Expression of gene involved in proliferation in ovary after PD-MSC transplantation. The expression of protein of pAkt, PI3K, pGSK3β, pFOXO3a and capspase-9 in ovary tissue isolated from OVX rats at 1, 2, 3 and 5 weeks after PD-MSCs transplantation were analyzed by Western blot (n=5 per group). GAPDH was used as an internal control. All experiments were performed in triplicate. [file 13287_2020_2002_MOESM3_ESM.tif]

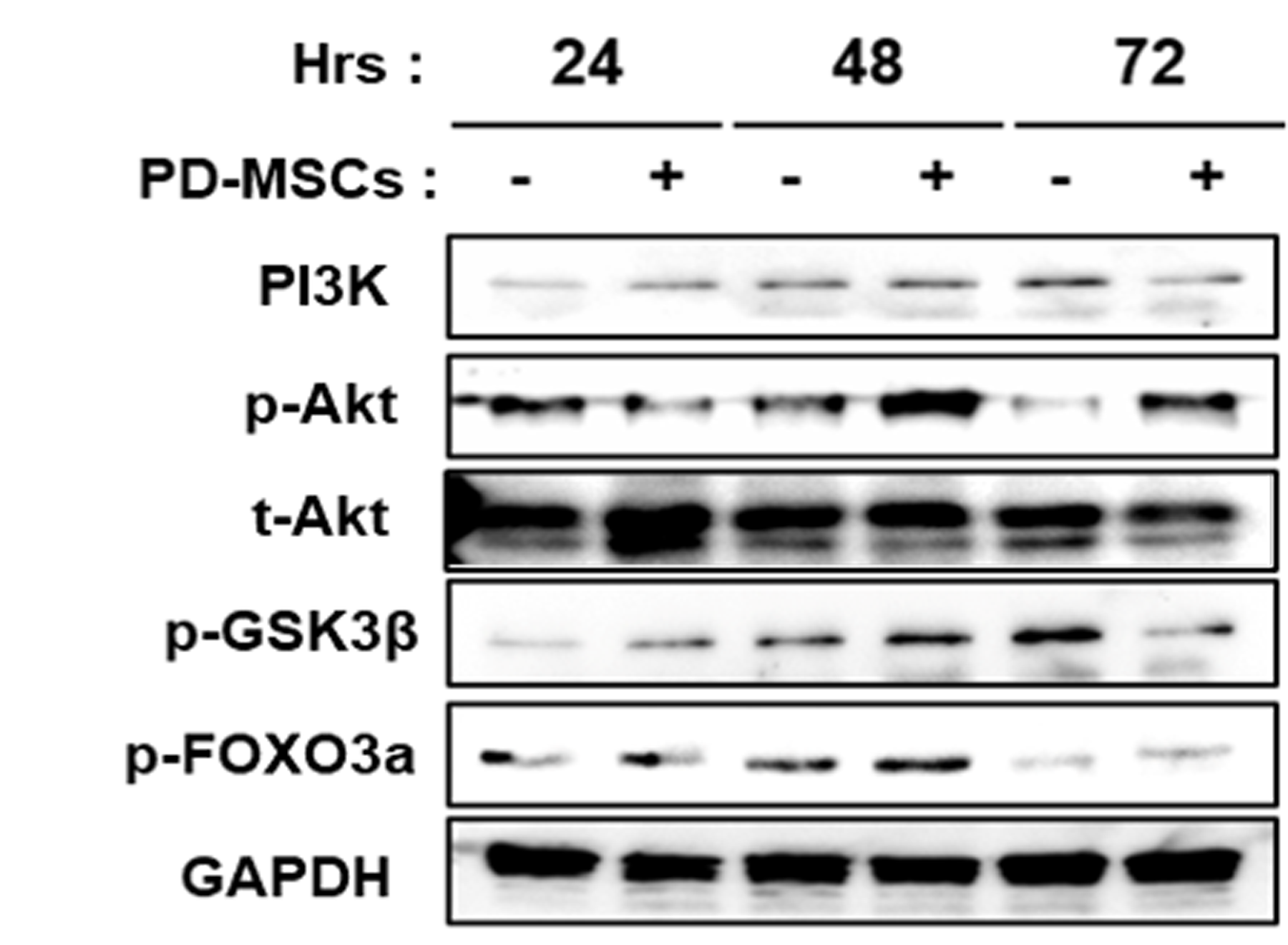

Supplement: Supplementary file 4 — Additional file 4 : Figure S3. [file 13287_2020_2002_MOESM4_ESM.tif]

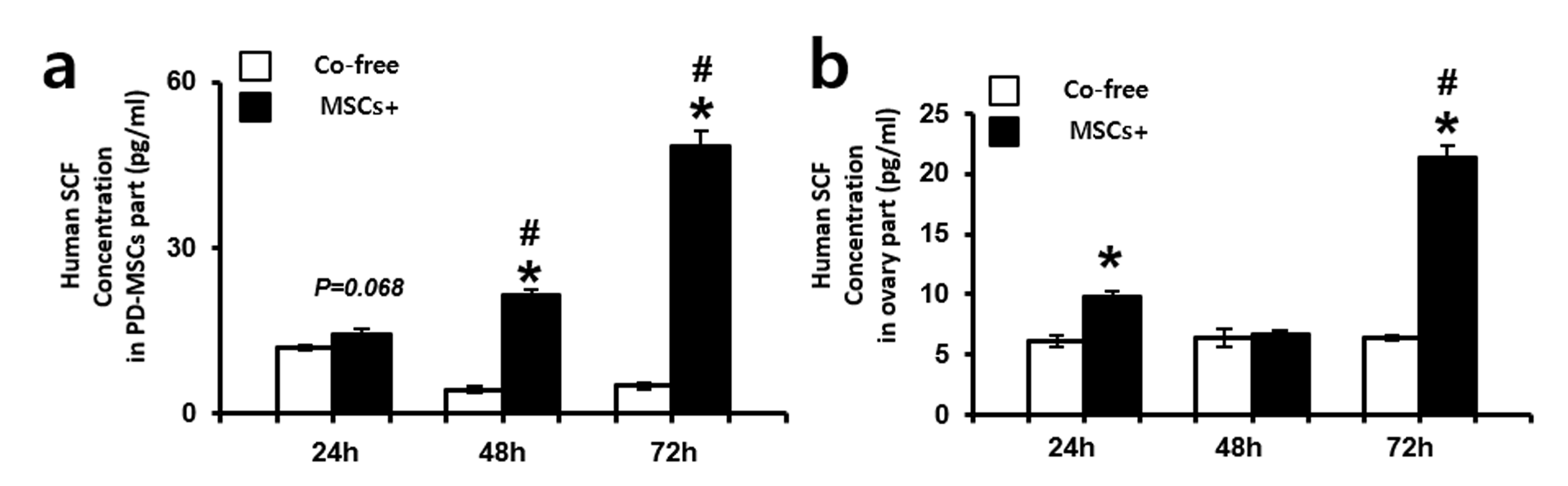

Supplement: Supplementary file 5 — Additional file 5 : Figure S4. [file 13287_2020_2002_MOESM5_ESM.tif]
